# Construction of a Bivalent Thrombin Binding Aptamer and Its Antidote with Improved Properties

**DOI:** 10.3390/molecules22101770

**Published:** 2017-10-19

**Authors:** Quintin W. Hughes, Bao T. Le, Grace Gilmore, Ross I. Baker, Rakesh N. Veedu

**Affiliations:** 1 Western Australian Centre for Thrombosis and Haemostasis, Discovery Way, Murdoch University, Perth, WA 6150, Australia; g.gilmore@wacth.org (G.G.); ross@haemwest.com.au (R.I.B.); 2 Perth Blood Institute, Hollywood Private Hospital, Monash Avenue, Perth, WA 6009, Australia; 3 Centre for Comparative Genomics, Discovery Way, Murdoch University, Perth, WA 6150, Australia; T.Le2@murdoch.edu.au; 4 Perron Institute for Neurological and Translational Science, Perth, WA 6009, Australia

**Keywords:** modified nucleotide, aptamer, thrombin binding aptamer, triethylene glycol linkage

## Abstract

Aptamers are short synthetic DNA or RNA oligonucleotides that adopt secondary and tertiary conformations based on Watson–Crick base-pairing interactions and can be used to target a range of different molecules. Two aptamers, HD1 and HD22, that bind to exosites I and II of the human thrombin molecule, respectively, have been extensively studied due to their anticoagulant potentials. However, a fundamental issue preventing the clinical translation of many aptamers is degradation by nucleases and reduced pharmacokinetic properties requiring higher dosing regimens more often. In this study, we have chemically modified the design of previously described thrombin binding aptamers targeting exosites I, HD1, and exosite II, HD22. The individual aptamers were first modified with an inverted deoxythymidine nucleotide, and then constructed bivalent aptamers by connecting the HD1 and HD22 aptamers either through a triethylene glycol (TEG) linkage or four consecutive deoxythymidines together with an inverted deoxythymidine nucleotide at the 3′-end. The anticoagulation potential, the reversal of coagulation with different antidote sequences, and the nuclease stability of the aptamers were then investigated. The results showed that a bivalent aptamer RNV220 containing an inverted deoxythymidine and a TEG linkage chemistry significantly enhanced the anticoagulation properties in blood plasma and nuclease stability compared to the existing aptamer designs. Furthermore, a bivalent antidote sequence RNV220AD efficiently reversed the anticoagulation effect of RNV220 in blood plasma. Based on our results, we believe that RNV220 could be developed as a potential anticoagulant therapeutic molecule.

## 1. Introduction

In 1990, two reports described the isolation of short single-stranded oligonucleotide sequences that can bind to a target molecule with high affinity and specificity [1,2]. The process described was referred to as the Systematic Evolution of Ligands by Exponential Enrichment (SELEX), which uses oligonucleotide sequence libraries (~10^15^–10^18^ members) to eventually isolate sequences called aptamers that bind to a selected target molecule with high affinity and specificity [3,4]. The high affinity of the selected aptamer is due to its ability to adopt unique secondary and tertiary structure dictated by Watson–Crick base-pairing interactions. Since discovery, aptamer research has exploded with nearly 7000 aptamer-related papers published across a wide range of fields—including medicine, biology, forensics, chemistry, counterterrorism, food safety, and the environment—with aptamers targeted towards an array of different molecules [5,6,7,8,9].

One of the most well studied aptamers is HD1, an aptamer targeting the exosite I moiety of thrombin (an important haemostatic protein) that was first described in 1992 [10]. Thrombin is central to the blood coagulation process, cleaving fibrinogen to fibrin, which forms the basis of a blood clot, amongst a number of other important activation steps [11]. The discovery of HD1 was soon followed by the development of a second thrombin targeting aptamer in 1997, HD22, which targets exosite II of the thrombin molecule [12]. Both aptamers exhibit anticoagulant activity, but HD1 exhibits a greater inhibition of fibrinogen cleavage due to the importance of exosite I in that mechanism [13]. The potential of HD1 and HD22 chimeras was also investigated, which increases the anticoagulant effect, strengthening the feasibility of using a bivalent form as a potential future therapeutic [14,15,16,17,18]. However, it should be noted that initial interest in the therapeutic potential of aptamers has waned in recent years after several clinical trials, including two RNA-based aptamers targeting coagulation factors, activated factor IX (REG1; Regado Biosciences, later merged with Tobira Therapeutics and recently acquired by Allergan plc, Dublin, Ireland) and von Willebrand factor (ARC1779; Archemix Inc. Cambridge, MA, USA), and a DNA-based aptamer targeting a tissue factor pathway inhibitor (BAX499; Baxter International Inc., Deerfield, IL, USA), were terminated for a range of issues including serious anaphylactic reactions and bleeding. Another impediment to therapeutic aptamer applications is the issue of ensuring long-term stability and viability of aptamers in the circulation due to the action of nucleases. Incorporation of chemically modified nucleotides such as locked nucleic acids (LNAs) and other modified nucleotides into aptamers during or after SELEX, or the introduction of polyethylene glycol (PEG) can be used to improve the serum stability and bio-availability [19,20,21,22,23,24]. A comprehensive review about chemical modifications of thrombin binding aptamer can be found elsewhere [25,26]. Herein, we investigate the potential of chemically modified HD1, HD22 and bivalent chimeras to determine the effect on the anticoagulant profile and the reversibility using complementary antidote (AD) sequences, and their stability to nuclease degradation.

## 2. Results

### 2.1. The Effect of Introducing Chemically Modified Nucleotides into the HD1 and HD22, and the Assessment of Bivalent Anti-Thrombin Aptamer Designs

**Figure 1:**
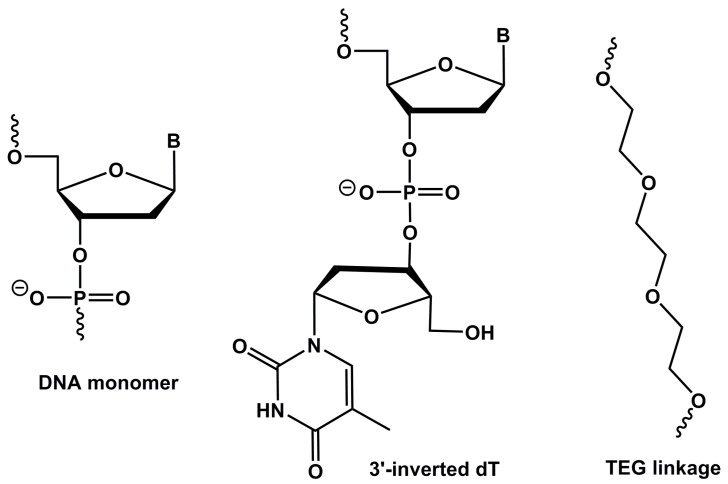
Structural representation of DNA, 3′-inverted monomer and triethylene glycol (TEG) linkage used in this study. B, nucleobase.

**Figure 2:**
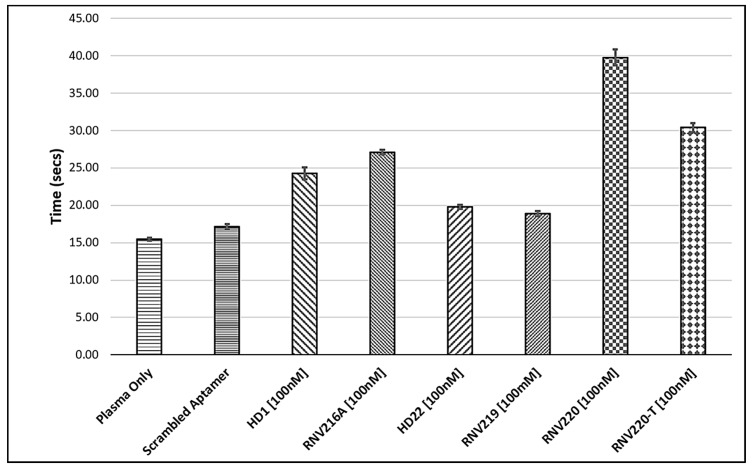
Thrombin clotting time (TCT) analysis of the aptamers RNV216A, RNV219, RNV220, and RNV220-T.

First, we constructed the modified variants of the original HD1 and HD22 aptamers by incorporating an inverted dT (inv-dT, Figure 1) nucleotide at the 3′-end position primarily to increase the stability to exonuclease degradation (RNV216A, modified HD1; RNV219, modified HD22; Table 1) and evaluated anticoagulant properties by measuring the thrombin clotting time (TCT). To further prolong the clotting time, we also made bivalent chimeras by linking the exosite I and exosite II binding aptamers using triethylene glycol (TEG, Figure 1; RNV220; Table 1) and by using four consecutive deoxythymidines (RNV220-T; Table 1), and with an inv-dT at the 3′-end to increase nuclease resistance. TCT analysis was performed using reconstituted normal blood plasma standard at a 100 nM concentration and the clotting time was recorded in seconds. The modified HD1, RNV216A, with an inv-dT residue, marginally increased the TCT, albeit not significantly (Figure 2), whereas the modified HD22, RNV219, did not show any improvement in the TCT values. Interestingly, the bivalent aptamer chimeras RNV220 and RNV220-T significantly improved the TCT in comparison to the scrambled control sequence in comparison with other tested aptamers. Remarkably, RNV220 with a TEG linker was found to be the most efficient molecule with a TCT of 39.75 s compared to RNV220-T containing four consecutive dT linkers with a TCT of 30.4 s.

### 2.2. Evaluation of the Reversal of Thrombin Clotting Using the Antidote Sequences

**Figure 3:**
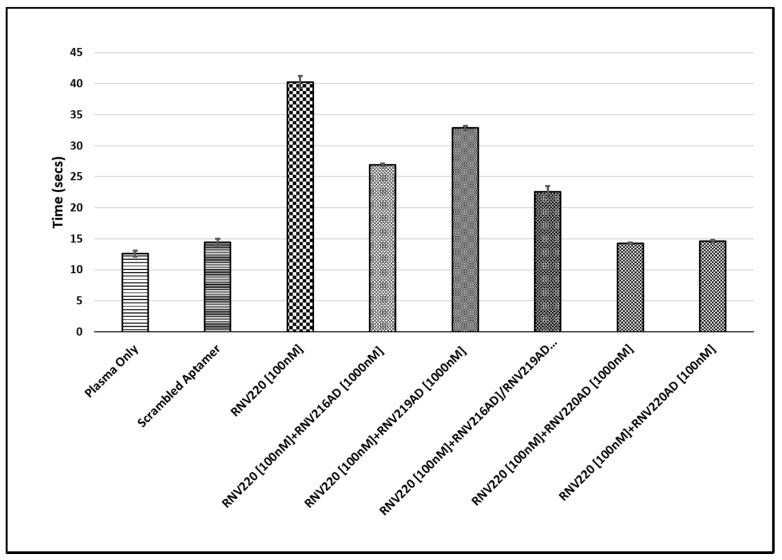
TCT analysis of the antidote sequences RNV216AD, RNV219AD, and RNV220AD to the aptamer RNV220.

Next, we investigated the reversal of anticoagulant effect using antidote sequences. As we found that the bivalent chimeric aptamer RNV220 containing a TEG linker and inv-dT had the highest TCT values, we used this molecule to investigate the efficacy of antidote sequences to reverse the anticoagulant effect. We constructed three different antidote sequences, RNV216AD, RNV219AD, and RNV220AD (Table 1). In this assay, antidote sequences were added to plasma containing 100 nM RNV220 at 1000 nM and/or 100 nM concentrations, 5 min prior to initialising the reaction. TCT analysis was performed and the clotting time was recorded in seconds. In this study, we used the antidote sequences RNV216AD (targeting HD1) and RNV219AD (targeting HD22) to RNV220 alone and in combination, in parallel to a full-length complementary antidote sequence RNV220AD with a TEG linker covering both regions, and the anticoagulant aptamer RNV220 as a control. Our results showed that the anticoagulant effect could not be completely reversed in the presence of 10-fold excess (1000 nM) of either RNV216AD or RNV219AD (Figure 3). Even, a combination RNV216AD and RNV219AD at 1000 nM concentration could not return the TCT to baseline levels observed for the 100 nM Scrambled control (Figure 3), suggesting that the use of individual aptamer antidotes targeting exosite I and II, respectively, may not be an efficient approach. Remarkably, we found that RNV220AD, a bivalent chimeric antidote molecule linked via TEG, efficiently reversed the anticoagulant effect of RNV220 at 1000 nM, and surprisingly the effect was prominent even at an equal concentration (100 nM) of the aptamer RNV220 (Figure 3).

### 2.3. Nuclease Stability Analysis of the Thrombin Binding Aptamers

**Figure 4:**
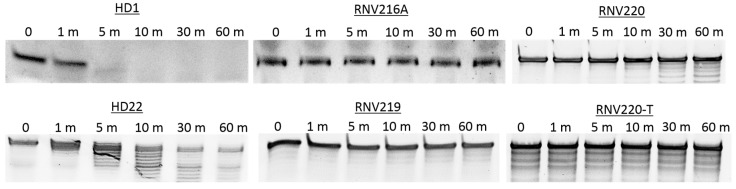
Nuclease stability analysis of the aptamers used in this study. (m = minute).

High nuclease stability is critical if an aptamer is to be transitioned to clinical development. In line with that, the primary aim of this study was to investigate whether specific modifications to the aptamer design could increase resistance to nuclease degradation, whilst maintaining a strong anticoagulant activity. The stability of all tested anticoagulant aptamers (Table 1) was investigated using snake venom phosphodiesterase, a harsh enzyme with very high 3′ → 5′ exonuclease activity. The aptamers were incubated with the enzyme at 37 °C, and the samples were collected at different time points (0, 1, 5, 10, 30 and 60 min). Products were then analysed on a 20% denaturing polyacrylamide gel, stained with SYBR Gold staining dye and visualised under UV light. The results clearly showed that HD1 and HD22 aptamers without the 3′ inv-dT modification degraded quickly within 10 min of incubation (Figure 4). On the other hand, RNV216A, RNV219, RNV220, and RNV220-T showed very high stability to phosphodiesterase attack even after 60 min of incubation, highlighting the importance of inv-dT incorporation at the 3′-end (Figure 4). The same trend was also observed when the aptamers were exposed to human serum for 0, 0.5, 1, 2, 4 and 6 h (Figure S1; Supplementary Information).

## 3. Discussion

Nucleic acid aptamer technology has attracted significant attention in therapeutic and diagnostic development since its invention in 1990. Thrombin binding aptamers (TBAs) are one of the most studied forms of aptamers because of their anticoagulant properties. However, the TBAs failed to meet the clinical expectations during the clinical trial stages due to suboptimal dosing profiles and poor pharmacodynamics properties. Since then, research has shifted toward the improvement of TBAs for developing efficient anticoagulant drugs as potential treatment options for the growing numbers of thrombotic complications worldwide. In this study, we designed and evaluated the efficacy of chemically modified anti-thrombin aptamers targeting both exosite I and/or exosite II, based on previously reported DNA aptamers HD1 (binding exosite I) and HD22 (binding exosite II). We recently reported that the incorporation of an inv-dT substantially improved the resistance to nuclease degradation [27]. In line with this data, we modified the aptamers HD1 and HD22 with an inv-dT (Figure 1) incorporation at the 3′-end and constructed RNV216A and RNV219, respectively, and evaluated the anticoagulation efficacy with a TCT assay in blood plasma. RNV216A, the modified exosite I binding aptamer, showed a slight improvement in TCT with 27 s compared to the reported HD1 (23 s). However, the modified exosite II binding RNV219 did not show any improvement (Figure 2). Müller et al. reported a bivalent design of TBA by linking HD1 and HD22 using poly-dA nucleotide linker [14]. Early this year, Pica et al. showed that the binding of HD1 to thrombin increases the affinity of HD22 aptamer to exosite II [16]. In addition, we recently reported the modification of HD1 using a carbon spacer molecule (C3-spacer) and found a significant increase in TCT [28]. Stemming from this work, in this study, we constructed two bivalent chimeric aptamers using exosite I binding HD1 and exosite II binding HD22 aptamers with two different linkers such as TEG (RNV220) and poly-dT (RNV220-T) containing an inv-dT at the 3′-end. Both the aptamers showed an increase in TCT by more than 2-fold compared to the scrambled, however, RNV220 was found to be the best (39.75 s) compared to RNV220-T (30.4 s). We speculate that this may be due the flexibility of the TEG moiety on the chimera.

Reversal of anticoagulation also important clinically, and the development of the antidote sequences to the most efficient anti-thrombin aptamers are necessary. RNV220, being the most efficient TBA, was analysed for TCT in the presence of antidote sequences complementary to HD1 (RNV216-AD) and HD22 (RNV219AD) aptamer regions, in addition to using a TEG-linked complementary HD1 and HD22 antidote sequence, RNV220-AD. The individual addition of RNV216AD or RNV219AD failed to fully reverse the RNV220-treated TCT. Notably, the combined addition of RNV216AD and RNV219AD also could not completely nullify the anticoagulation effect of RNV220 even at a higher dose of 1000 nM (10-fold excess). Interestingly, the addition of RNV220AD completely reversed the anticoagulation effect of RNV220, highlighting the significance of a bivalent design for developing an efficient antidote sequence. It was also noted that the effect was very prominent even at an equal dose (100 nM) of RNV220.

Nuclease stability of all modified TBAs were analysed in the presence of snake venom phosphodiesterase with very high 3′ → 5′ exonuclease activity. The results were not surprising as HD1 and HD22 composed of natural nucleotide showed poor resistance against exonuclease degradation. However, all the aptamers modified with an inv-dT at the 3′-end (RNV216A, RNV219, RNV220, and RNV220-T) were highly stable even after 1 h of incubation (Figure 4), highlighting the potential of inv-dT nucleotides to improve the pharmacokinetic properties. In general, all modified aptamers used in this study except RNV219 showed improved anticoagulation properties compared to their natural counterparts, which may in part be due to the improved stability by inv-dT incorporation.

## 4. Materials and Methods

### 4.1. Aptamer Design and Synthesis

All aptamers used in this study were sourced commercially (IDT, Coralville, IA, USA), and all sequences are detailed in Table 1.

### 4.2. Thrombin Clotting Time (TCT) Assay

The TCT assay was performed on a Sysmex CS-5100 (Siemens Healthineers, Erlangen, Germany) using reconstituted pooled normal commercial plasma (Diagnostica Stago, Paris, France). Aptamer was added to produce final concentrations of either 1000 nM or 100 nM in 490 μL of commercial plasma (final volume 500 μL). Aptamers were pre-incubated in the plasma for 5 min prior to initiation of TCT and clotting times recorded in seconds. To test the antidote sequences test aptamers were again added 5 min prior to the addition of test antidote sequences that were added immediately before the initiation of the TCT reaction.

### 4.3. Nuclease Degradation Assay

Two micromolar concentrations of HD1, HD22, RNV216A, RNV219, RNV220, and RNV220-T were incubated with 0.00002 U of the phosphodiesterase enzyme from *Crotalus adamanteus* venom (Sigma Aldrich, St. Louis, MO, USA) at 37 °C. The reaction was quenched at different time points—0, 1 min, 5 min, 10 min, 30 min and 1 h, by adding 8 μL of formamide loading buffer in equal volume of the reaction mixture. The reaction mixture was then separated on a 20% denaturing polyacrylamide gel. The gel was stained with SYBR Gold (Thermo Fisher Scientific, Waltham, MA, USA) for 5 min before visualising under UV light using a Fusion Fx gel documentation system (Vilber Lourmat, Marne-la-Vallee, France).

### 4.4. Human Serum Degradation Assay

Five micromolar concentrations of HD1, HD22, RNV216A, RNV219, RNV220, and RNV220-T were incubated with human serum at 37 °C. The reaction was quenched at different time points—0, 0.5, 1, 2, 4, and 6 h—by adding 8 μL of formamide loading buffer in an equal volume of the reaction mixture. The reaction mixture was then separated on a 15% denaturing polyacrylamide gel. The gel was stained with SYBR Gold (Thermo Fisher Scientific, Waltham, MA, USA) for 5 min before visualising under UV light using Fusion Fx gel documentation system (Vilber Lourmat, Marne-la-Vallee, France).

## 5. Conclusions

In conclusion, we have developed novel thrombin binding aptamers with improved anticoagulant properties by modifying the previously reported thrombin binding aptamers targeting thrombin exosite I and exosite II domains. Construction of a bivalent chimeric aptamer using TEG linkage chemistry showed the highest thrombin clotting time in human plasma. Likewise, the triethylene-glycol-linked chimeric bivalent complementary antidote was found to be very effective in the reversal of the anticoagulation effect of the modified TBA. Based on our results, we firmly believe that the further development of RNV220 and RNV220AD may be very useful in developing potent therapeutics in tackling thrombotic disorders.

## Figures and Tables

**Table 1:** Aptamer and antidote sequences used in our analysis. The chemical modifications are underlined.

NAME	SEQUENCE (5′-3′)
HD1	GGT TGG TGT GGT TGG
RNV216A	GGT TGG TGT GGT TGG/inv-dT
HD22	AGT CCG TGG TAG GGC AGG TTG GGG TGA CT
RNV219	AGT CCG TGG TAG GGC AGG TTG GGG TGA CT/inv-dT
RNV220	GGT TGG TGT GGT TGG /TEG/ AGT CCG TGG TAG GGC AGG TTG GGG TGA CT/inv-dT
RNV220-T	GGT TGG TGT GGT TGG /TTTT/ AGT CCG TGG TAG GGC AGG TTG GGG TGA CT/inv-dT
RNV216-AD	CCA ACC ACA CCA ACC
RNV219-AD	AGT CAC CCC AAC CTG CCC TAC CAC GGA CT
RNV220-AD	AGT CAC CCC AAC CTG CCC TAC CAC GGA CT /TEG/ CCA ACC ACA CCA ACC

## Supplementary Materials

Supplementary Materials are available online. Figure S1: Human serum degradation assay of the aptamers used in this study.
