# Supplementary material for: Construction of a Bivalent Thrombin Binding Aptamer and Its Antidote with Improved Properties
Source: Molecules. 2017 Oct 19;22(10):1770. doi: 10.3390/molecules22101770 (PMC6151750; doi:10.3390/molecules22101770)
Supplement: Supplementary file 1 [file molecules-22-01770-s001.pdf]

Construction of a bivalent thrombin binding aptamer and its antidote with improved properties.

Quintin W. Hughes [1,2]*, Bao T. Le [3,4], Grace Gilmore [1,2], Ross I. Baker [1,2] and Rakesh N. Veedu [3,4]*

1 Western Australian Centre for Thrombosis and Haemostasis, Discovery Way, Murdoch University, Perth, WA 6150, Australia.
2 Perth Blood Institute, Hollywood Private Hospital, Monash Avenue, Perth, WA 6009, Australia.
3 Centre for Comparative Genomics, Discovery Way, Murdoch University, Perth, WA 6150, Australia.
4 Perron Institute for Neurological and Translational Science, Perth, WA 6009, Australia.
* Correspondence: R.Veedu@murdoch.edu.au; q.hughes@wacth.org; Tel.: +61-8-9360-2803

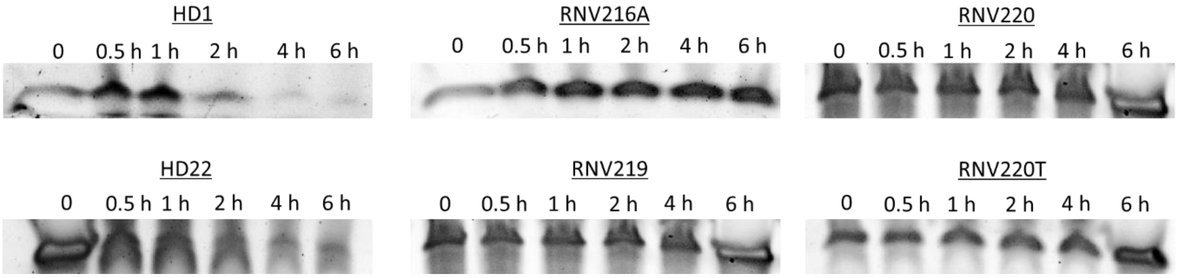

Figure S1. Human serum degradation assay of the aptamers used in this study. (h = hour).
